# PD-1 pathway-mediated regulation of islet-specific CD4^+^ T cell subsets in autoimmune diabetes

**DOI:** 10.14800/ie.1164

**Published:** 2016

**Authors:** Tijana Martinov, Justin A. Spanier, Kristen E. Pauken, Brian T. Fife

**Affiliations:** Center for Immunology, Department of Medicine, Division of Rheumatic and Autoimmune Diseases, University of Minnesota, Minneapolis, MN, 55455, USA

**Keywords:** type 1 diabetes, CD4^+^ T cells, insulin, anergy, PD-1, checkpoint blockade

## Abstract

Type 1 diabetes (T1D) is a CD4^+^ T cell-driven autoimmune disease resulting from the destruction of insulin-producing pancreatic beta cells. Clinical evidence and studies in non-obese diabetic (NOD) mice suggest that insulin is a major autoantigen. With this in mind, we developed insulin B_10-23_:IA^g7^ tetramer reagents to track insulin-specific CD4^+^ T cells in mice and interrogated the role of Programmed death-1 (PD-1) for peripheral tolerance. PD-1 is a T cell inhibitory receptor necessary to maintain tolerance and prevent T1D in NOD mice. PD-1 pathway inhibitors are increasingly used in the clinic for treating malignancies, and while many patients benefit, some develop adverse autoimmune events, including T1D. We therefore sought to understand the role of PD-1 in maintaining islet-specific tolerance in diabetes-resistant strains. B6.g7 mice express the same MHC Class II allele as NOD mice, have predominantly naïve insulin-specific CD4^+^ T cells in the periphery, and remain diabetes-free even after PD-1 pathway blockade. Here, we examined the trafficking potential of insulin-specific CD4^+^ T cells in NOD and B6.g7 mice with or without anti-PD-L1 treatment, and found that PD-L1 blockade preferentially increased the number of CD44^high^CXCR3^+^ insulin-specific cells in NOD but not B6.g7 mice. Additionally, we investigated whether pancreatic islets in NOD and B6.g7 mice expressed CXCL10, a lymphocyte homing chemokine and ligand for CXCR3. Anti-PD-L1 treated and control NOD mice had detectable CXCL10 expression in the islets, while B6.g7 islets did not. These data suggest that islet tolerance may be in part attributed to the pancreatic environment and in the absence of pancreas inflammation, chemotactic cytokines may be missing. This, together with our previous data showing that PD-1 pathway blockade preferentially affects effector but not anergic self-specific T cells has implications for the use of checkpoint blockade in treating tumor patients. Our work suggests that determining tumor- and self-specific CD4^+^ T cell activation status (naïve, effector or anergic) prior to initiation of immunotherapy would likely help to stratify individuals who would benefit from this therapy versus those who might have adverse effects or incomplete tumor control.

## Introduction

Type 1 diabetes (T1D) is caused by the immune-mediated destruction of insulin-producing pancreatic beta cells in the islets of Langerhans ^[1]^. An estimated 3 million people currently suffer from T1D in the United States alone. In the last decade, incidence has risen by 23% among individuals younger than 20 years of age, and this alarming trend is expected to continue ^[2]^. Daily insulin injections are the standard of care, but they are not a cure. Due to artificial blood glucose regulation, T1D patients remain at an increased risk of heart and kidney disease, blindness and peripheral neuropathy ^[3–5]^. These complications have a significant impact on the quality of life and longevity ^[6]^. Islet transplantation is an attractive therapeutic approach, but requires immunosuppression. Understanding how islet-reactive lymphocytes are activated, escape peripheral tolerance, and cause disease is necessary to design antigen-specific therapies to cure T1D.

Clinical evidence as well as studies using the non-obese diabetic (NOD) mouse model of spontaneous T1D demonstrate that CD4^+^ and CD8^+^ T cells are critical for beta cell destruction ^[7–13]^. While a case study described T1D onset in a patient with X-linked agammaglobulinemia ^[14]^, new-onset T1D patients benefited from B cell depletion therapy, suggesting that B cells are also required for disease ^[15]^. In fact, B cell-mediated autoantibody production against islet antigens precedes T1D onset and is currently the only immunological biomarker of disease progression ^[3]^. Specifically, insulin autoantibody onset can predict time to overt T1D in mice ^[16]^ and to a lesser degree in humans. All patients who develop T1D before age 5 have insulin autoantibodies ^[17]^, suggesting that insulin is a critical autoantigen. In NOD mice, as many as 90% of insulin-specific CD4^+^ T cells target insulin B_10-23_ residue ^[18]^. This peptide is required for T1D, as a single mutation in a T cell receptor contact site abrogates disease ^[19]^. With that in mind, we and others developed insulin B_10-23_:MHC Class II tetramer reagents ^[20–23]^ to track insulin-specific CD4^+^ T cells during disease development and at onset in NOD mice, as well as interrogate the fate of this population in diabetes resistant B6.I-A^g7^ (B6.g7) mice to understand tolerance mechanisms in play ^[24, 25]^.

**Figure 1:**
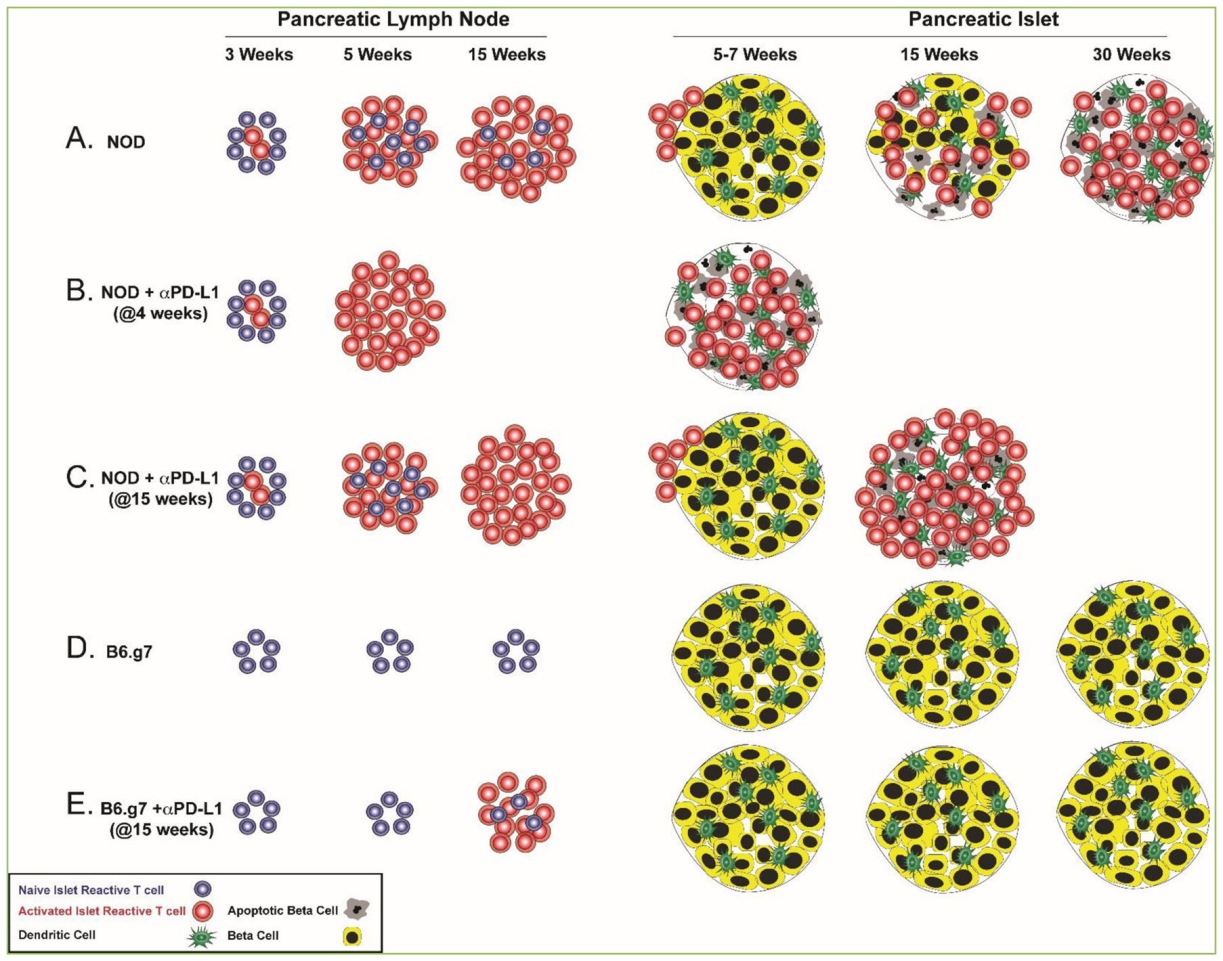
Model of islet antigen-specific CD4 T cell activation and islet inflammation timeline in NOD and B6.g7 mice (A) In NOD mice, insulin-specific CD4^+^ T cells encounter antigen in the pancreatic lymph node, expand and acquire the potential to traffic to the pancreas between 3 and 5 weeks of age. Pancreatic inflammation becomes more severe as mice age and peaks at disease onset, between 10 and 30 weeks (15 weeks as illustrated here). (B) Anti-PD-L1 treatment at 4 weeks of age significantly enhances T cell activation, thus leading to accelerated diabetes onset within 20 days in 90% of treated animals ^[38]^. (C) PD-1 pathway blockade at a later time point when existing insulitis is extensive (at 15 weeks for example) ^[25]^, leads to a very rapid disease onset in NOD mice within 1–3 days ^[38]^. (D) In B6.g7 mice, insulin-specific CD4^+^ T cells do not expand as mice age and ~80% of the islets remain uninfiltrated ^[25]^. (E) PD-1 pathway blockade at 15 weeks of age leads to an increased number of activated insulin-specific CD4^+^ T cells in the pancreatic lymph node, but insulin reactive T cells do not traffic to the pancreas and the extent of islet inflammation does not change ^[25]^.

Programmed death-1 (PD-1) is a T cell inhibitory receptor, and it is highly expressed on recently activated effector T cells as well as chronically stimulated (anergic) CD4^+^ and (exhausted) CD8^+^ T cells, thus limiting their antiviral and antitumor activity ^[26–28]^. Blocking PD-1 signaling has the potential to reinvigorate anergic or exhausted cells. This spurred the development of PD-1 pathway inhibitors (checkpoint blockade) for the treatment of advanced malignancies ^[29, 30]^. While some patients benefit from this treatment, it is unclear why others do not, or why some patients develop adverse events and proceed to develop autoimmune-like symptoms or overt autoimmunity, including T1D ^[31]^. PD-1 SNPs have been shown to increase the risk of T1D development in several populations ^[32–35]^, suggesting that at least in a subset of patients, PD-1 plays a critical role in maintaining islet tolerance. Deficiency in, or blocking PD-1 from interacting with its ligand programmed death ligand-1 (PD-L1), accelerates T1D onset in NOD mice (Figure 1); ^[36–40]^). However, the role PD-1/PD-L1 pathway plays in islet tolerance maintenance in non-autoimmune prone mouse strains is unclear. To address this gap in knowledge, we sought to determine whether the PD-1 signaling pathway regulated islet-specific CD4^+^ T cells in mice of varying autoimmune susceptibilities, as well as whether anergy maintenance required continuous PD-1 signaling ^[25]^.

To illustrate previous work combined with results presented here, we have generated a model figure depicting the relative number of antigen specific cells, expansion, trafficking to the pancreas, and islet destruction (Figure 1). Compared to NOD mice (Figure 1A), B6.g7 mice had a detectable population of insulin-specific CD4^+^ T cells in the secondary lymphoid organs (SLO), but these cells remained predominantly naïve throughout the lifetime of the animal (Figure 1D) and expressed low levels of PD-1^[25]^. PD-1 pathway blockade led to an increased number of activated insulin-specific CD4^+^ T cells in the pancreatic lymph node of both NOD (Figure 1B, C) and B6.g7 mice (Figure 1E), but B6.g7 mice remained disease- and infiltrate-free ^[25]^ (Figure 1E). PD-1/PD-L1 deficiency on a diabetes-prone background (NOD) led to accelerated disease as previously reported, and increased numbers of insulin-specific but not foreign antigen-specific CD4^+^ T cells in the SLO and pancreas ^[25]^.

Since the majority of islet-specific CD4^+^ T cells in NOD mice are anergic ^[24]^, we also investigated whether PD-1 pathway blockade restores function to these cells. Anergic cells were defined as cells producing little to no IFNγ and expressing CD73 and folate receptor 4 (FR4) on their surface ^[24, 41]^. Effector cells were defined as FR4^−^CD73^−^ cells capable of producing IFNγ following stimulation ^[24, 41]^. Following anti-PD-L1 treatment, effector cells produced significantly more IFNγ on a per cell basis. While PD-1 blockade led to a modest increase in the amount of IFNγ produced by anergic cells, IFNγ production was still blunted in comparison to effector cells ^[25]^. This suggested that PD-1 pathway blockade preferentially impacted the effector cell subset, and did not uniformly break T cell anergy.

In the current study, we evaluated the insulin-specific CD4^+^ T cell trafficking potential from NOD and B6.g7 mice with and without PD-1 pathway blockade. Collectively, our findings suggest that PD-1 blockade was not sufficient to result in B6.g7 lymphocyte homing to the pancreas due to lower expression of CXCR3 on insulin-specific CD4^+^ T cells, and an absence of a CXCL10 chemotactic gradient.

## Materials and Methods

### Mice

NOD mice were purchased from Taconic (Germantown, NY). B6.g7, NOD.PD-1^−/−^ (PD-1 KO), and NOD.PD-L1^−/−^ (PD-L1 KO) mice were generated as described ^[25, 42]^ and housed in specific-pathogen free conditions at the University of Minnesota. All animal experiments were approved by the Institutional Animal Care and Use Committee.

### PD-1 pathway blockade

9 or 15 week old NOD and B6.g7 mice were treated with 250μg anti-PD-L1 (clone MIH6; ^[42]^) or isotype control (Rat IgG2a; BioXCell) at day −3 and −1 prior to harvest for CXCR3 and CXCL10 analysis.

### Detection of antigen-specific T cells

Insulin-specific CD4^+^ T cells were detected using insB_10-23r3_:I-A^g7^ tetramer reagent and dual color staining with magnetic enrichment as described ^[24, 25]^. Briefly, single cell suspensions were incubated for one hour at room temperature with 10 nM of tetramers conjugated to phycoerythrin (PE) and allophycocyanin (APC) in medium containing Fc receptor block (2.4G2) and 0.05% sodium azide. Spleen and non-draining lymph node samples were subjected to magnetic enrichment following a 30 minute incubation with both anti-PE and anti-APC microbeads at 4°C (Miltenyi Biotec). Single cell suspensions from the pancreas were isolated using collagenase P digestion (Roche) and discontinuous Percoll gradients (44%/67%) ^[24, 25]^. Following tetramer staining, single cell suspensions from pancreas and pancreatic lymph node were subjected to surface staining for flow cytometric analysis.

### Flow cytometry

Staining was performed for 30 minutes at 4°C using anti-PD-1 (J43), CD4 (GK1.5), CD3 (145-2C11), B220 (RA3-6B2), CD11b (M1/70), CD11c (N418), CD44 (IM7), CXCR3 (CXCR3-173) (eBioscience) and anti-CD8α (53-6.7) antibodies (Biolegend). Samples were acquired using BD LSRII or Fortessa instruments (BD) and analyzed with Flow Jo software (Treestar). Insulin-specific CD4^+^ T cells were identified as singlet^+^, CD3^+^ lineage (B220, CD11b, CD11c)^−^ CD4^+^ CD8α^−^, insB_10-23r3_: I-A^g7^-PE and -APC tetramer double positive ^[24, 25]^.

### Immunofluorescence Microscopy

Pancreata were harvested for immunofluorescence and prepared as described ^[42]^. Antibodies included guinea pig anti-swine insulin (Dako, Denmark), goat anti-mouse CXCL10 (R&D systems), donkey anti-guinea pig AF488 and bovine anti-goat AF647 (Jackson Immunoresearch), and hamster anti-mouse CD3-PE (eBioscience). Slides were mounted using Prolong Gold with DAPI (Life Technologies) and imaged on a Leica epifluorescent DM5500 microscope (Germany).

## Results and Discussion

Emerging results from genome wide association studies point to PD-1 as a critical risk factor in T1D pathogenesis ^[32–35]^. Additionally, PD-1 pathway inhibitors used to treat advanced malignancies have led to T1D development in several patients, indicating that at least in a subset of individuals, PD-1 is required for restraining islet-reactive T cells ^[31]^. Increasing use of checkpoint blockade therapies warrants a better understanding of how PD-1 regulates islet-reactive CD4^+^ T cells in contexts of varying autoimmune susceptibilities. To this end, we examined insulin-specific CD4^+^ T cells following checkpoint blockade in diabetes-prone and diabetes-resistant mice expressing the same MHC class II molecule.

**Figure 2:**
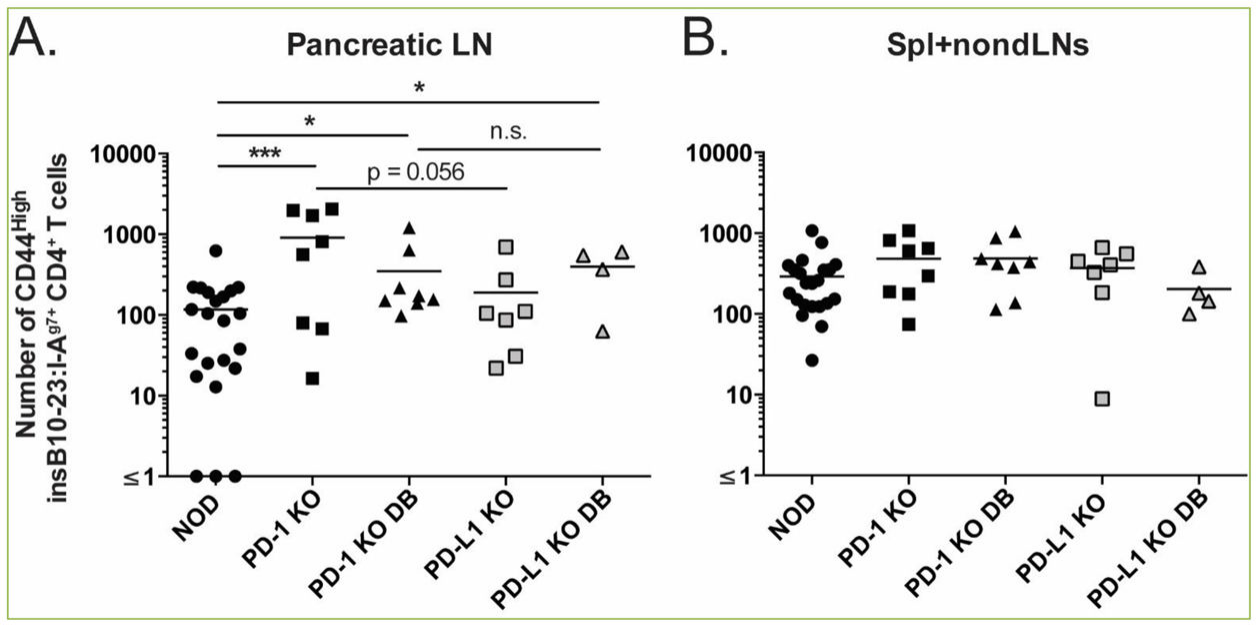
NOD PD-1/PD-L1 deficiency leads to increased activation of insulin-specific CD4^+^ T cells The frequency of activated (CD44^high^) insulin specific T cells was determined from (A) pancreatic lymph nodes and (B) Spleen and non-draining lymph nodes of 5–6 week old PD-1 KO (n=8), PD-L1 KO (n=4–7), and age-matched littermate NOD controls (n=22) by flow cytometry prior to or at disease onset (“DB”=diabetes) using insB_10-23r3_:I-A^g7^ tetramer reagents. Shown are compiled data from four independent experiments.

Genetic loss of PD-1 or PD-L1 in NOD mice led to an increased number of insulin-specific but not foreign antigen-specific CD4^+^ T cells ^[25]^. Not only was there an increase in the abundance of insulin-specific cells in both PD-1 KO and PD-L1 KO mice, these cells exhibited increased expression of the activation marker CD44 compared to age-matched NOD mice (Figure 2). The number of CD44^high^ insulin-specific CD4^+^ T cells was significantly higher in the pancreatic lymph nodes of prediabetic (907.9±310.4 cells) and diabetic PD-1 KO mice (347.2±137.0 cells) (p<0.001 and p<0.01, respectively) compared to prediabetic NOD mice (117.0±29.5 cells) (Figure 2A). Similarly, diabetic PD-L1 KO mice had a significantly greater number of CD44^high^ insulin-specific CD4^+^ T cells (395.9±122.2 cells) compared to age-matched NOD mice (p <0.01) (Figure 2A). The number of activated insulin-specific CD4^+^ T cells in the spleen and non-draining lymph nodes (spl+non-dLNs) was not different across these strains (Figure 2B). Additionally, CD44 expression on foreign antigen-specific CD4^+^ T cells was comparable (data not shown).

**Figure 3:**
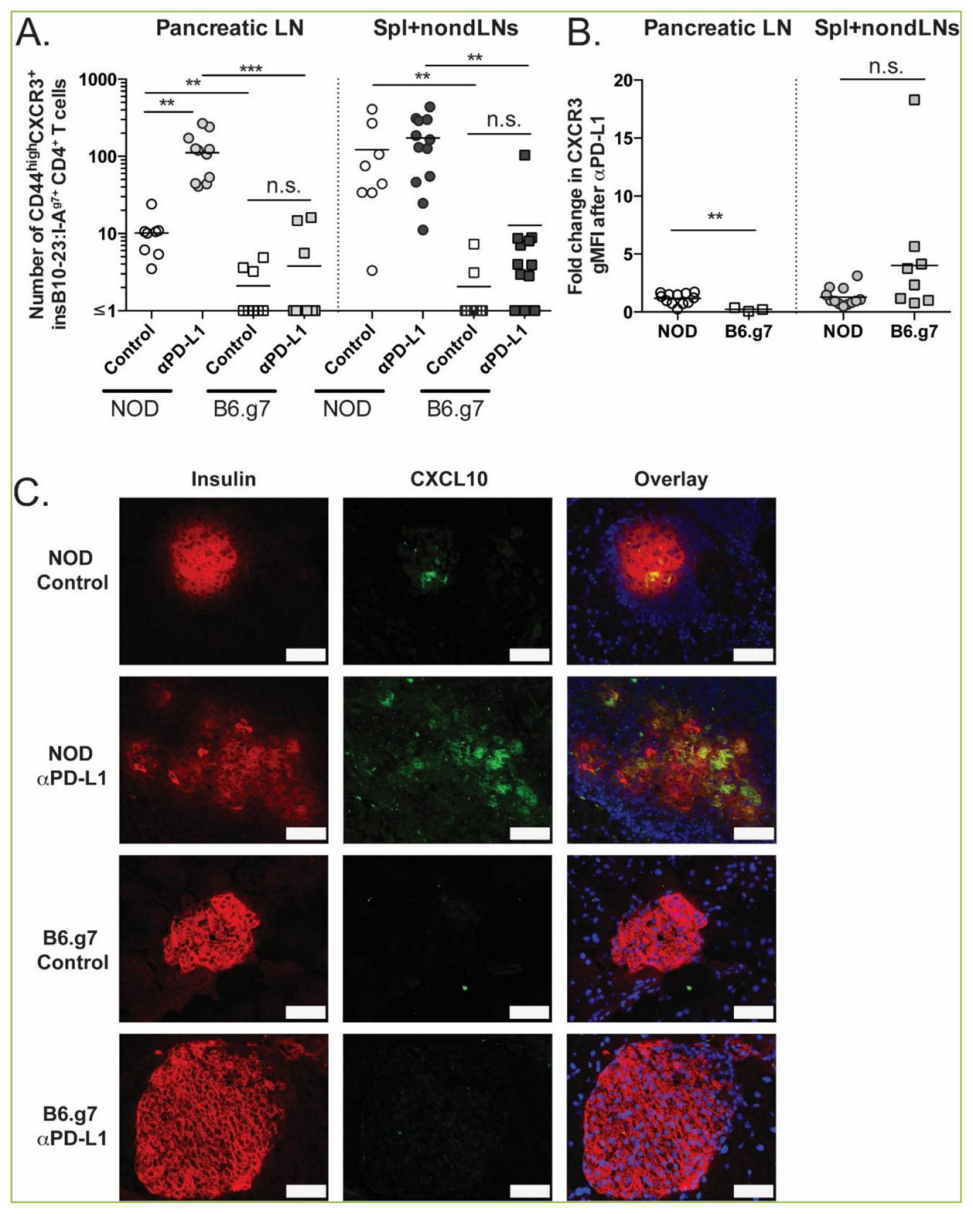
PD-1 pathway blockade increases the number of CD44^high^ CXCR3^+^ insulin-specific CD4^+^ T cells in NOD but not B6.g7 mice NOD and B6.g7 mice were treated at day −3 and −1 with anti-PD-L1 (MIH6 clone; 250 μg/mouse) or isotype control (Rat IgG2a; 250 μg/mouse). (A) The number of CD44^high^CXCR3^+^ insulin-specific CD4^+^ T cells (identified by double-staining with insB_10-23r3_: I-A^g7^-tetramers as described in Methods) in the secondary lymphoid organs of control and anti-PD-L1-treated NOD and B6.g7 mice. (B) Fold increase in geometric mean fluorescent intensity (gMFI) of CXCR3 after PD-L1 blockade from insulin-specific CD4^+^ T cells. Shown are compiled data from four independent experiments with n=2–3 per treatment group. (C) Immunofluorescent staining of CXCL10 expression in pancreas tissue from NOD and B6.g7 mice from anti-PD-L1 or isotype treated animals. Scale bar (white) corresponds to 50 μm. Images are representative of three independent experiments with >100 islets/treatment group.

PD-1 pathway blockade led to an increase in the number of activated insulin-specific CD4^+^ T cells in the pancreatic lymph nodes of NOD and B6.g7 mice ^[25]^ (Figure 1B, E), but B6.g7 mice remained insulitis- and diabetes-free ^[25]^ (Figure 1E). We sought to determine whether anti-PD-L1 treatment altered the trafficking potential of insulin-specific cells in NOD or B6.g7 mice. Pancreas homing is regulated by the CXCR3-CXCL10 axis ^[43, 44]^. Therefore, we assessed the expression levels of CXCR3 on insulin-specific CD4^+^ T cells in NOD and B6.g7 mice that were treated with anti-PD-L1 or control at days -3 and -1 prior to harvest. Anti-PD-L1 led to a significant increase in the number of CD44^high^CXCR3^+^ cells in the pancreatic lymph nodes of NOD (10.16±2.25 vs. 111.5±23.7, p<0.01), but not B6.g7 mice (2.09±0.55 vs. 3.78±1.62) (Figure 3A). Additionally, the number of insulin-specific CD4^+^ T cells with the potential to traffic was higher in NOD pancreatic lymph nodes compared to B6.g7 in control (p <0.01) and anti-PD-L1 treated mice (p<0.001). While anti-PD-L1 treatment did not lead to a significant increase in CD44^high^CXCR3^+^ cells in NOD spleen and nondraining lymph nodes, the number of cells with trafficking potential was larger than in B6.g7 mice (p<0.01 between control and anti-PD-L1 treated mice). The fold increase in the geometric mean florescence intensity (gMFI) of CXCR3 was higher on CD44^high^ insulin-specific cells after anti-PD-L1 treatment in the pancreatic lymph node of NOD mice (p<0.01) (Figure 3B). These results suggest that NOD islet-reactive cells were more susceptible to PD-1 pathway blockade allowing enhanced activation (CD44) and greater ability to traffic to tissues with inflammation including the pancreas (CXCR3).

The inflammatory cytokine interferon gamma (IFNγ) is known to be important for disease onset ^[45, 46]^. CXCL10, or interferon regulated protein of 10Kd, is a chemokine that is made in response to IFNγ and inflammation ^[43, 47]^. We hypothesized that B6.g7 islets lacked chemotactic gradients necessary to recruit the autoreactive cells. To test this, we examined islet CXCL10 expression by histology to correlate the level of islet infiltration with chemokine production. CXCL10 was absent from B6.g7 islets even after PD-L1-blockade, while CXCL10 was readily detectible in the islets of age-matched treated and untreated NOD mice (Figure 3C). Collectively, these findings indicate that there are at least two levels of islet tolerance: T cell-intrinsic and pancreas-microenvironment specific. We hypothesize that the initial wave of islet inflammation depends on CCL2, CCL5, and CXCL9 secreted in response to inflammatory cytokines such as IL-1β and TNF-α ^[48]^, and that CCR2^+^ or CCR5^+^ T cell-derived IFNγ drives CXCL10 expression in pancreatic beta cells to recruit CXCR3^+^ insulin-specific T cells. Further studies are needed to determine whether altering the pancreatic environment in B6.g7 mice would be sufficient to allow for T cell infiltration, islet destruction and diabetes development.

Our previous work suggests that the susceptibility to PD-1 blockade-mediated functional restoration depends on the differentiation state of CD4^+^ and CD8^+^ T cells ^[25]^. Effector, but not anergic autoreactive CD4^+^ and CD8^+^ T cells were reinvigorated after PD-1 blockade, suggesting that blocking PD-1 signaling alone is not sufficient to rescue effector function for all anergic cells ^[25]^. Therefore, blocking the PD-1/PD-L1 pathway alone might not be sufficient to reinvigorate all anergic tumor-specific cells, but it also might not be sufficient to restore full effector cell function of previously anergic, autoreactive T cells and/or allow T cell homing to target organ(s). Determining tumor- and self-specific CD4^+^ T cell activation status prior to initiation of checkpoint blockade therapy would likely help to stratify individuals who would benefit from this therapy versus those who might have adverse effects or incomplete tumor control.

## Abbreviations

T1D: type 1 diabetes
NOD: non-obese diabetic
PD-1: programmed death-1
PD-L1: programmed death-ligand 1
SLO: secondary lymphoid organs
FR4: folate receptor 4.
